# Analyzing pediatric department documentation of patient social needs with ICD-10 Z-codes

**DOI:** 10.1186/s12913-026-14758-x

**Published:** 2026-06-03

**Authors:** Oluwatofunmi Jummie Akinwunmi, Deron Galusha, Baylah Hooper, Carol Oladele, Marcella Nunez-Smith, Karen H. Wang, Cecelia Calhoun

**Affiliations:** 1 Yale School of Public Health, New Haven, Connecticut, USA; 2 Equity Research and Innovation Center, Section of General Internal Medicine, Department of Medicine, Yale School of Medicine, New Haven, CT USA; 3 Section of Hematology, Department of Medicine, Yale School of Medicine, New Haven, Connecticut, USA

**Keywords:** Social needs, Social determinants of health, Z-code, Pediatric, ICD-10, Health system

## Abstract

**Background:**

The 10th revision of the International Classification of Diseases (ICD-10) allows healthcare professionals to document social needs with ICD-10 Z-codes. Data derived from the documentation of ICD-10 Z-codes can be leveraged to identify at-risk populations for targeted intervention. However, there remains concern for underutilization of these codes, particularly in the pediatric population. We sought to examine the documentation of ICD-10 Z-codes in the pediatric outpatient setting of the largest Connecticut health system by specialty type and age group.

**Methods:**

In this cross-sectional study, we examined deidentified electronic health records of pediatric outpatient patients seen within the Yale New Haven Health System in Connecticut from 2019 to 2020. We used descriptive statistics to measure the proportion of patients with at least one social needs-related ICD-10 Z-code. We compared the rates of documentation across different specialty types and age groups. We also identified nine categories of ICD-10 Z-codes and compared differences in the distribution of Z-code categories across age groups.

**Results:**

A total of 1,164 out of 59,867 pediatric outpatient patients (1.9%) from 2019 to 2020 had at least one documented ICD-10 Z-code. Across all ages, the specialty type with the highest rate of documentation was school-based health centers (10.6%), followed by pediatric primary care (5.3%). Within the pediatric primary care group, we found that patients aged 22 to 30 were significantly more likely than all other age groups to have an ICD-10 Z-code documented.

**Conclusions:**

We demonstrate that social needs documentation with ICD-10 Z-codes in the pediatric department of this health system is low overall and likely unreflective of the true social needs of the population. These findings suggest the need for further study of how pediatric social needs are identified, documented, and operationalized within clinical workflows.

**Supplementary Information:**

The online version contains supplementary material available at 10.1186/s12913-026-14758-x.

## Introduction

Health-related social needs (HRSNs)—such as housing instability, food insecurity, transportation barriers, and economic hardship—are major drivers of health outcomes and health inequities across the lifespan. These needs are particularly consequential when they occur in childhood, as early-life social adversity is strongly associated with poorer health, educational attainment, and economic outcomes in adulthood [1–3]. Despite this, HRSNs are inconsistently identified and documented in routine clinical care, limiting health systems’ ability to address them in a systematic and equitable manner.

Social needs identification and documentation is particularly important for the future health of pediatric patients. Childhood socioeconomic status (SES) is a powerful predictor of adult cardiovascular morbidity and mortality, and early exposure to adversity is strongly linked to learning, behavior, and mental wellbeing [1, 4]. In a review of 40 studies, Galobardes et al. found that adults with low childhood SES experience a higher risk of premature mortality, even when adult SES is accounted for [5, 6]. Large-scale work by Raj Chetty and colleagues further demonstrates that childhood neighborhood conditions, income inequality, and access to opportunity have profound and lasting effects on adult health, income, and life expectancy [7, 8]. Importantly, these effects reflect structural income disparities as children from lower-SES backgrounds are more frequently exposed to adverse social circumstances than their more affluent peers, resulting in cumulative disadvantage over the life course [9]. These findings underscore the importance of early identification and intervention to reduce the long-term health consequences of childhood social adversity.

Several recently implemented screening and referral programs have illustrated the role of social needs identification in addressing poor health outcomes. Programs such as the Trinity Health Innovative Healthcare Associates Program and Baylor Scott & White Health’s Community Advocate Program have shown that systematic screening for social needs enables connection to community-based resources and supportive services [10, 11], with the latter associated with reduced 30-day readmission rates of enrolled patients by over 85% [11]. At the same time, evidence from the Camden Coalition suggests that identification of social and medical complexity alone may be insufficient to improve utilization outcomes without broader supportive infrastructure [12]. Together, these findings suggest that while social needs identification is an important first step, its value depends in part on how that information is documented and used to support intervention.

In pediatric care, several practices have begun incorporating social needs screening into routine workflows. One such model, Well Child Care, Evaluation, Community Resources, Advocacy, Referral, Education (WE CARE), involves systematic screening for HRSNs during well-child visits, with referrals offered when families express interest in assistance [13, 14]. Screening tools such as WE CARE can identify unmet social needs at the point of care and support clinician-family discussions. However, because screening tools and documentation practices vary across clinics and health systems, these approaches do not consistently generate structured data that can be leveraged for longitudinal analysis at the system level.

One approach to capturing HRSNs is documentation within electronic health records through administrative billing codes. The 10th revision of the International Classification of Diseases (ICD-10) allows healthcare professionals to document social and economic circumstances with ICD-10 Z-codes. In addition to the larger body of ICD-10 codes used to document patient symptoms, diseases, and abnormal findings, these newer social needs-related Z-codes can be leveraged to identify at-risk populations and target them for specialized, preventive intervention [15]. Prior studies have found that patients with documented Z-codes have increased negative health events as well as higher healthcare utilization, total annual healthcare costs, and readmission rates [16–18]. These findings highlight the strong association between Z-code documentation and poor health outcomes.

Despite growing interest in integrating health-related social needs into clinical care, an important gap remains in understanding how social needs are documented in pediatric outpatient settings. Prior studies have shown that ICD-10 Z-codes are underutilized overall [18–20], but it remains unclear how frequently these codes are used in pediatric populations and whether documentation differs across pediatric specialty types and age groups. This gap is important because without structured documentation, health systems may miss opportunities to identify children and young adults with unmet social needs, track patterns across care settings, and develop targeted interventions. In this study, we examined the use of social needs-related ICD-10 Z-codes across pediatric outpatient practices within the largest integrated health system in Connecticut from 2019 to 2020. By characterizing documentation patterns by specialty type and age group, we aimed to identify gaps in current documentation practices and inform future system-level strategies to improve the identification of pediatric social needs.

## Methods

### Study design

We conducted a cross-sectional study using deidentified electronic health record (EHR) data from the Yale New Haven Health System in Connecticut, which includes five hospitals and over 100 outpatient clinics. This study population consisted of all individuals aged 0 to 30 years old who had at least one outpatient pediatric encounter from January 1st, 2019, to December 31st, 2020. A pediatric outpatient encounter was defined as any ambulatory visit billed under a pediatric specialty or pediatric primary care clinic within the health system. By limiting the scope of this study to one health system and focusing on the pediatric population from 2019 to 2020, we aimed to assess the needs of this specific population in Connecticut and identify departments within the health system that could benefit from targeted intervention.

The deidentified dataset used in this study included patient-level demographic variables (race, ethnicity, preferred language, legal sex, and age at the time of the outpatient encounter), outpatient specialty type, and documentation of ICD-10 Z-codes. Additional data such as diagnostic ICD-10 codes and health insurance type were not available in this dataset. Patients up to age 30 years old were included to capture young adults receiving care in pediatric continuity or specialty clinics.

The primary unit of analysis was the patient level. However, because some patients had encounters with multiple outpatient specialties, analyses stratified by specialty type were conducted at the patient–specialty level.

### Primary outcome

The primary outcome was the prevalence of documented HRSNs defined as the presence of at least one social needs–related ICD-10 Z-code per patient. We focused primarily on ICD-10 codes Z55-Z65, which have previously been identified as the codes most explicitly related to health-related social needs [17, 18]. The study also includes codes Z74.8 and Z91.89 because of their relation to transportation needs (“assistance needed with transportation” and “lack of access to transportation” respectively).

These Z-codes were grouped into nine categories in accordance with the ICD-10 classifications: education, housing and economic circumstances, employment, food security, occupational exposure, upbringing, social environment, psychosocial circumstances, and transportation (which included codes Z74.8 and Z91.89 as described above). The housing and economic category reflects the structure of ICD-10, which combines these domains. The upbringing category included codes related to child welfare custody, history of abuse or neglect, disruption of family by separation or divorce, and parent–child estrangement. A complete list of included Z-codes and their corresponding categories is provided in Appendix 1.

### Independent variables

The independent variables of interest included age and specialty type. Age was collapsed into 6 categories based on patient age at the time of the encounter: 0–2 (infant/toddler), 3–5 (preschool), 6–11 (grade school), 12–17 (teen), 18–21 (transition aged youth), and 22–30 (young adult). These categorizations were derived from the American Academy of Pediatrics [21]. Clinical sites were grouped into categories of “specialty types” based on the predominant form of care delivered at each site. The specialty types included pediatric primary care, pediatric subspecialty, and School-Based Health Centers (SBHCs). In this health system, SBHCs provide comprehensive healthcare to children from preschool to 12th grade (primarily serving children from 3 to 18 years) which includes acute care, routine health assessments and immunizations, psycho-social evaluations, counseling, nutrition support, medication management, laboratory testing, crisis intervention, and referral for specialty care [22]. Although SBHCs primarily serve children aged 3 to 18 years, a small number of observations in our dataset fell outside this range and were retained in the analysis because they were recorded within SBHC encounters in the health system data. Individuals could be assigned to more than one specialty type if they were patients of multiple specialty types within the study period.

Other variables of interest included race, ethnicity, and sex. Race and ethnicity were categorized using the classifications available in the electronic health record at the time of data capture (2019–2020), which were consistent with Office of Management and Budget (OMB) Statistical Policy Directive No. 15 (1997), the federal standard in effect during the study period [23]. Race was categorized as American Indian or Alaska Native, Asian, Black or African American, Native Hawaiian or Other Pacific Islander, Other/Not Listed, and White or Caucasian. Race categories with small cell counts (*n* < 11) were combined into the ‘Other/Not Listed’ category to ensure stability of estimates and avoid reporting sparse data. Ethnicity was categorized as Hispanic or Latino, Not Hispanic or Latino, or Unknown. Sex was encoded as a binary variable (male, female) in the dataset derived from the EHR.

### Analysis

We calculated the prevalence rate of documentation of social needs-related Z-codes, stratified by age group and specialty type. The chi-squared test with Yates continuity correction was used to compare documentation rates across groups. Given the exploratory nature of subgroup analyses, p-values were not adjusted for multiple comparisons and should be interpreted cautiously. Data processing was conducted in R, and visualization was conducted in both R and Microsoft Excel. All data analysis was conducted in 2023.

## Results

Of the sample of 59,867 pediatric outpatient patients from 2019 to 2020, 1164 (1.9%) had at least 1 documented ICD-10 Z-code (Table 1).

**Table 1:** Patient-level demographic characteristics of pediatric outpatient patients with and without documented ICD-10 Z-Codes

	Patients with Z-codes	Patients without Z-codes	P value
**Sample Size- n**	1164	58,703	
**Age Group- n (%)**			< 0.001
0 to 2	210 (18.0)	10,731 (18.3)	
3 to 5	175 (15.0)	8156 (13.9)	
6 to 11	286 (24.6)	14,941 (25.5)	
12 to 17	336 (28.9)	17,256 (29.4)	
18 to 21	118 (10.1)	6146 (10.5)	
22 to 30	39 (3.3)	1473 (2.5)	
**Race – n (%)**			< 0.001
Asian	58 (5.0)	1849 (3.1)	
Black or African American	264 (22.7)	10,390 (17.7)	
White or Caucasian	286 (24.6)	28,200 (48.0)	
Other/Not Listed	556 (47.8)	18,264 (31.1)	
**Ethnicity – n (%)**			< 0.001
Hispanic or Latino	670 (57.6)	19,015 (32.4)	
Not Hispanic or Latino	480 (41.2)	37,367 (63.7)	
Unknown	14 (1.2)	2321 (4.0)	
**Sex – n (%)**			0.42
Male	560 (48.1)	30,172 (51.4)	
Female	604 (51.9)	28,530 (48.6)	

Characteristics of pediatric outpatient patients with and without ≥ 1 documented ICD-10 Z-code. This table is reported at the patient level, with each patient counted once. The “Other/Not Listed” category includes “American Indian or Alaska Native” and “Native Hawaiian or Other Pacific Islander” categories, which were combined due to small cell counts (*n* < 11 in at least one comparison group). Values are presented as n (%) unless otherwise indicated. *P* values reflect comparisons between groups

**Fig. 1:**
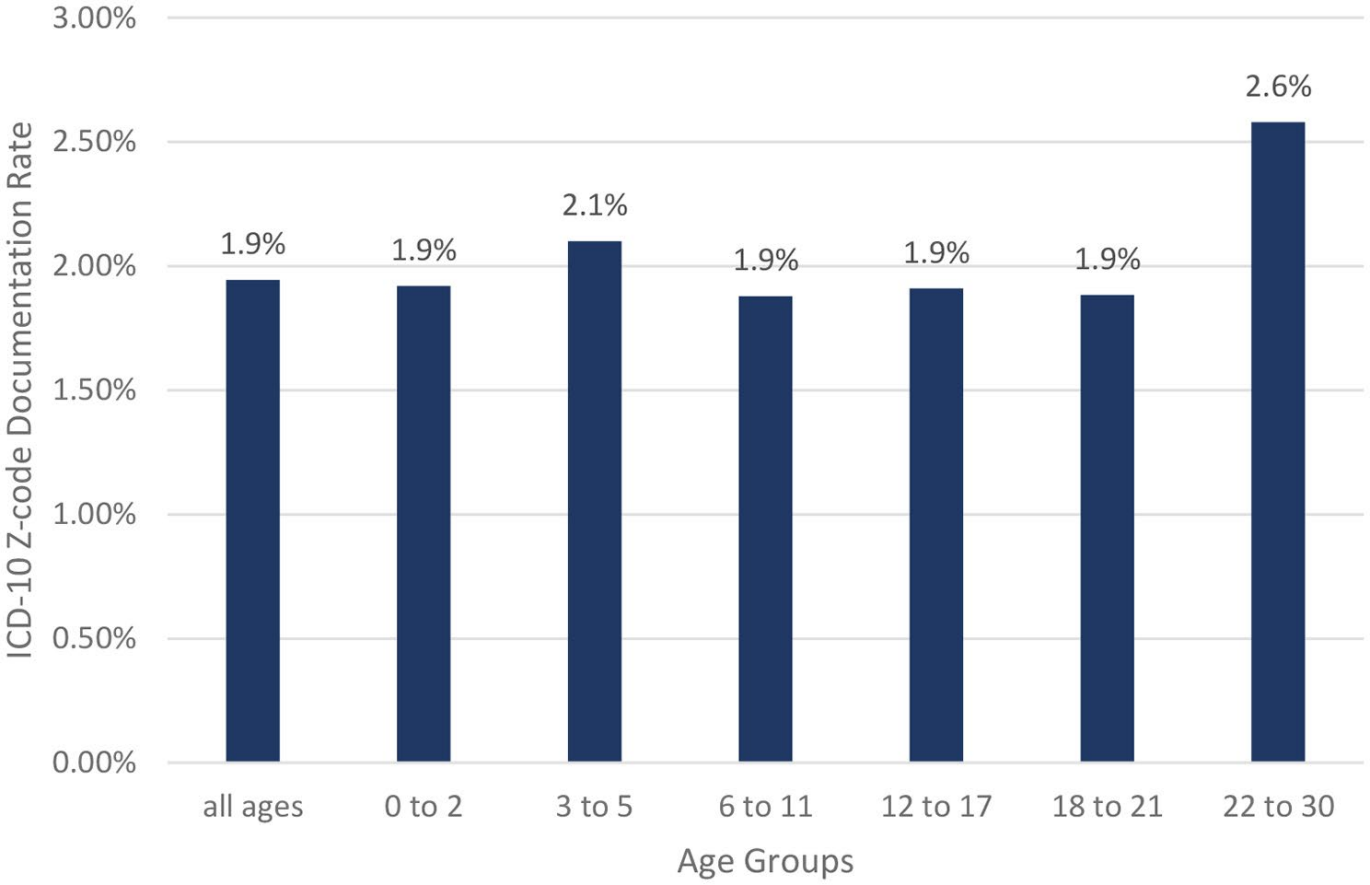
ICD-10 Z-code Documentation Rate by Age Group. Proportion of pediatric outpatient patients with ≥ 1 documented ICD-10 Z-code, stratified by age group

Across all pediatric specialty types, patients in the 22 to 30 age group were more likely to have Z-codes documented when compared to all other age groups; however, this difference was not found to be statistically significant (2.6% vs. 1.9%; *P* = 0.086) (Table 1; Fig. 1). White patients were significantly less likely to have documented Z-codes when compared to all other racial groups (1.0% vs. 2.8%; *P* < 0.001). Hispanic patients were significantly more likely to have Z-codes documented than patients not Hispanic or Latino (3.5% vs. 1.3%, *P* < 0.001) (Table 1).

**Fig. 2:**
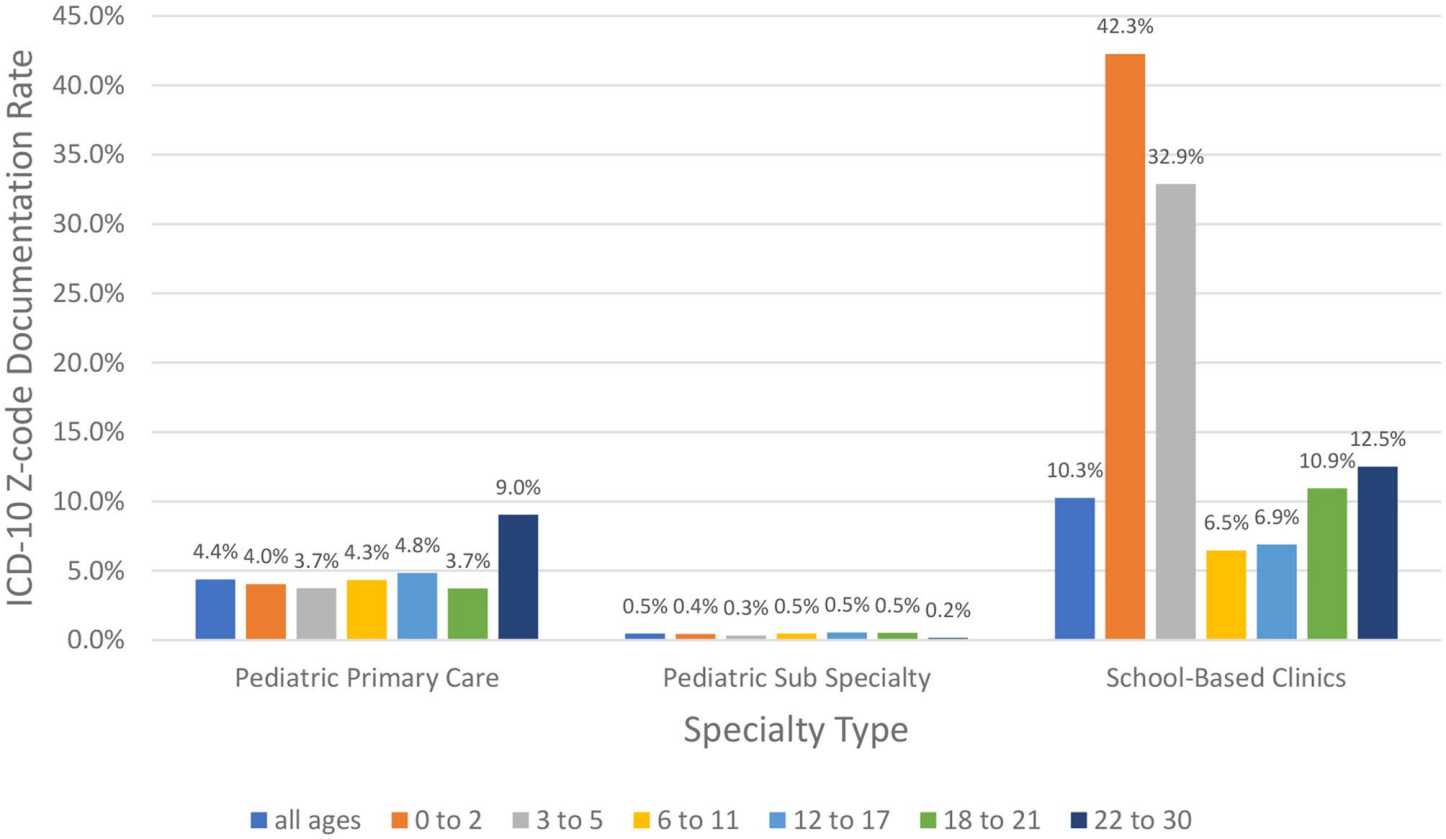
ICD-10 Z-code Documentation Rate by Specialty Type and Age Group. Proportion of pediatric outpatient patient-specialty observations with ≥ 1 documented ICD-10 Z-code, stratified by specialty type (pediatric primary care, pediatric subspecialty, and school-based clinics) and age group

When comparing documentation rates within the three specialty types, we found that patients seen in SBHCs were significantly more likely to have a documented Z-code when compared to those in the pediatric primary care setting (10.3% vs. 4.4%; *P* < 0.001). Z-codes were least likely to be documented in the pediatric subspecialty setting (0.5%). Among patients seen in SBHC, the highest rates of Z-code documentation occurred within the 0 to 2 age group (42.3%) and the 3 to 5 age group (32.9%) (Table 2; Fig. 2). Because the number of SBHC observations in the 0 to 2 age group was small, this finding should be interpreted cautiously. Among those in the pediatric primary care setting, we found that patients aged 22 to 30 were significantly more likely to have a Z-code documented than those in all other age groups (9.0% vs. 5.2%, *P* < 0.001) (Fig. 2).

**Table 2:** Distribution of ICD-10 Z-Code Documentation by Specialty Type at the Patient–Specialty Level

	Patients with Z-codes	Patients without Z-codes	P value
**Sample Size- n**	1164	58,703	
**Specialty Type – n (%)**			< 0.001
Pediatric primary care	745 (64.0)	16,320 (27.8)	
Pediatric subspecialty	214 (18.4)	45,600 (77.7)	
School-based health centers	228 (19.6)	1996 (3.4)	

Distribution of pediatric outpatient observations with and without ≥ 1 documented social needs–related ICD-10 Z-code across specialty types. This table is reported at the patient–specialty level; therefore, individual patients could contribute to more than one specialty category if they were seen in multiple clinic types during the study period. As a result, specialty counts may exceed the number of unique patients. Values are presented as n (%). *P* values reflect comparisons between groups

**Fig. 3:**
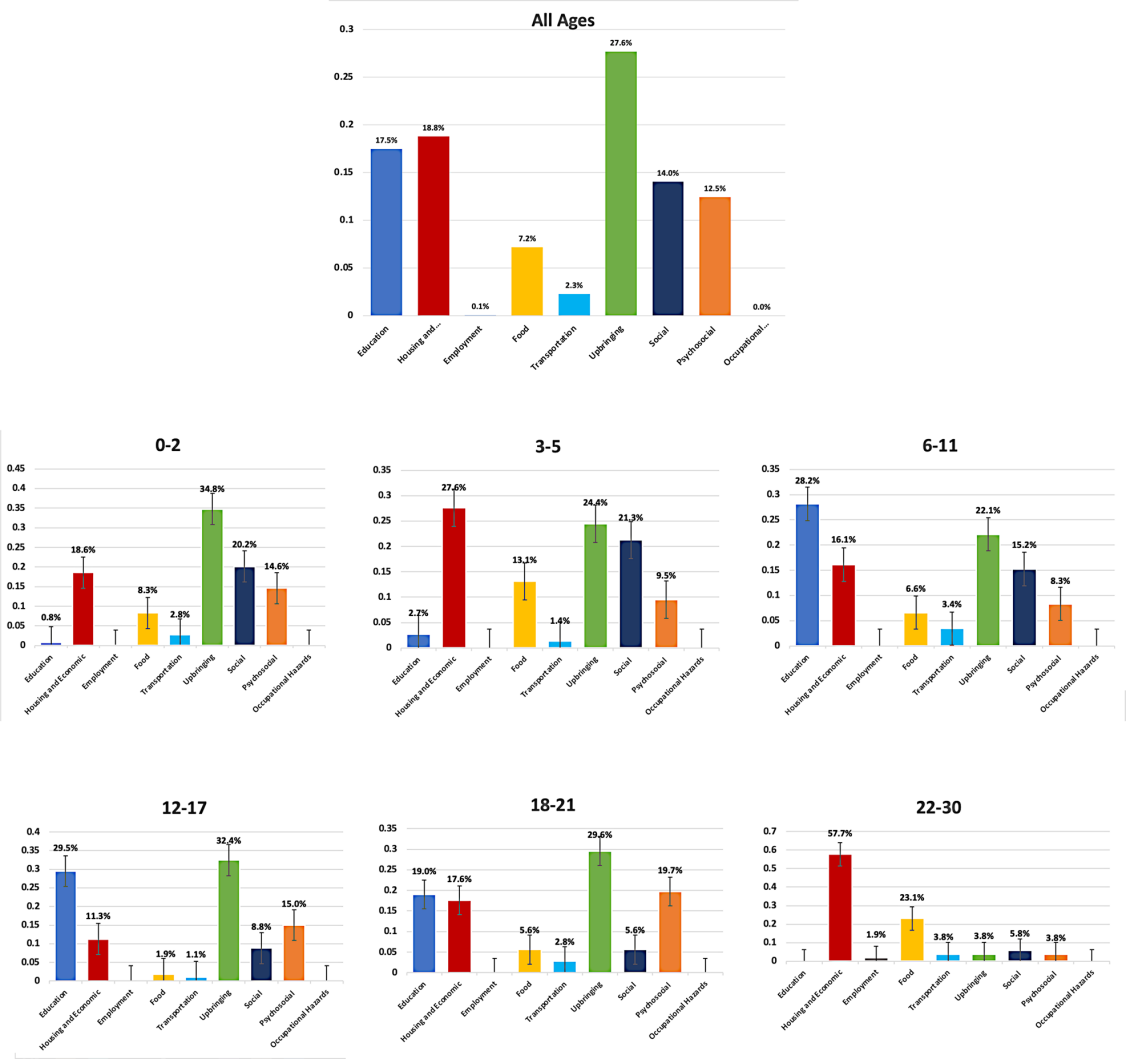
Types of documented ICD-10 Z-codes by age group. Distribution of documented ICD-10 Z-code categories among pediatric outpatient patient observations with ≥ 1 Z-code, stratified by age group, from 2019–2020. Categories are not mutually exclusive; patient were counted in multiple categories if more than one Z-code category was documented

Across all age groups, the most common types of Z-codes documented were those relating to upbringing as well as housing and economic circumstances. Z-codes relating to upbringing accounted for 28.5% of Z-codes in the 0 to 21 age group. This percentage was significantly greater than the percentage of upbringing-related Z-codes documented for those in the 22 to 30 age group (28.5% vs. 4.0%; *P* = 0.01). Conversely, Z-codes relating to housing and economic issues accounted for 57% of the Z-codes documented in the 22 to 30 age group, which was significantly greater than the proportion observed across all other age groups combined (57.0% vs. 17.3%; *P* < 0.001). Across all age groups, we found that the least common types of Z-codes were those relating to occupational exposure (0%), employment (0.07%), and transportation (2.3%) (Fig. 3).

## Discussion

In this study, we found that provider documentation of health-related social needs using ICD-10 Z-codes was low overall and varied substantially by clinical setting and patient age. Together, these findings highlight persistent gaps in the structured documentation of pediatric social needs and underscore a potential missed opportunity for health systems to identify and address social adversity early in the life course.

### Persistently low rates of Z-code documentation

Across all pediatric specialty types and age groups, only 1.9% of patients had a documented social needs–related Z-code. This finding is consistent with prior national and pediatric-specific studies that reported Z-code documentation rates between 1% and 5% [19, 24–26]. Despite this consistency, the prevalence remains strikingly low when compared to population-level measures of pediatric social needs.

During the study period, the overall poverty rate in Connecticut was 9.7%, and the poverty rate among young children (age < 5 years) was 14.6% [27]. In 2020, 12.6% of Connecticut children lived in food-insecure households [28]. Beyond material hardship, recent statewide estimates indicate that 12.3% of children in Connecticut have experienced two or more adverse childhood experiences, reflecting exposure to family instability, household substance use or mental illness, violence, discrimination, incarceration of a household member, or parental death [29].

Taken together, these population-level measures suggest a substantial gap between the prevalence of pediatric social needs in the community and the proportion documented within structured health system records, underscoring the likelihood that ICD-10 Z-code documentation markedly underrepresents true social need burden.

### Multilevel explanations for under-documentation with Z-codes

The low prevalence of Z-code documentation likely reflects barriers at multiple levels. At the patient level, it has been well described in the literature that families with greater social needs face structural barriers to accessing outpatient care, including transportation challenges, unstable housing, or competing caregiving responsibilities [30–32]. These barriers reduce their ability to attend outpatient visits and can thereby limit opportunities for their health-related social needs to be documented in the first place.

At the provider level, low levels of social needs-related ICD-10 Z-codes documentation may have reflected limited incentive to use Z-codes to document health-related social needs [24, 33]. More recently, CMS has increasingly emphasized the collection of health-related social needs and established payment for certain related services under the Physician Fee Schedule; however, ICD-10 Z-codes themselves are not separately reimbursable and are not directly linked to physician payment [34, 35]. In contrast to diagnostic ICD-10 codes, social needs-related Z-codes offered no immediate financial or administrative benefit, potentially reducing their uptake in busy clinical settings.

Furthermore, while medical providers are aware of the important impact of social needs on patient health [36], they may prefer to document social needs in free text clinical notes rather than through structured coding. Navathe et al. found that social needs were identified at significantly higher rates in clinical notes than in structured electronic health record data [37]. Narrative documentation has the benefit of allowing clinicians to capture nuance and context of a patient’s situation, which may be clinically meaningful for communication across a care team and for documenting complex psychosocial realities that may not map cleanly onto a single code. However, narrative documentation limits the ability of health systems to aggregate data, identify population-level trends, or implement standardized interventions across settings. This lack of standardization may perpetuate inequities, as patients receiving care in clinics without robust screening and documentation workflows may be less likely to be identified as having unmet social needs.

### Higher documentation in school-based health centers

We observed that SBHCs had a significantly higher rate of Z-code documentation than pediatric primary care and pediatric subspecialty settings. SBHCs play a critical role in increasing access to high quality, comprehensive, and coordinated primary care for underserved children and adolescents [38]. They often provide comprehensive primary care services that address the physical and mental health needs of students. Because of their unique position at the intersection of health and education, they have been shown to alleviate accessibility and financial barriers to consistent health care [38, 39]. Furthermore, providers who work in these settings may have greater exposure to social stressors affecting children and families, as well as more time and contextual information to assess social needs. The higher rates of Z-code documentation in SBHC may indicate that clinical settings that tend toward more comprehensive, whole-child care may be more equipped to incorporate documentation of social needs with ICD-10 Z-codes into their clinical workflow.

### Elevated documentation among young adults in pediatric care

Another notable finding was the higher prevalence of Z-code documentation among young adults aged 22 to 30 receiving care in pediatric primary care clinics. Prior work has shown that nearly half of commercially insured youth transition from pediatric- to adult-focused primary care later than the recommended 18–21 year age range. Furthermore, the American Academy of Pediatrics explicitly supports continued pediatric care beyond age 18 for patients with chronic conditions [40, 41]. Higher rates of Z-code documentation in this population may reflect greater social complexity, more frequent healthcare encounters, or longer-standing relationships with pediatric providers that facilitate identification of unmet social needs. Furthermore, young adults may be more likely to directly disclose housing, financial, transportation, educational, or psychosocial challenges during clinical encounters, whereas for younger children such needs may depend on parent or caregiver disclosure, which may be influenced by stigma, privacy concerns, fear of judgment, or uncertainty about the relevance of sharing these issues in a medical setting.

Importantly, this period of young adulthood during which patients transition from pediatric to adult care may represent a vulnerable transition during which failure to identify and address social needs may contribute to gaps in adult care engagement and adverse long-term outcomes [42, 43]. This vulnerable period is often marked by changes in care teams, insurance coverage, social support, and increasing responsibility for self-management, all of which can contribute to fragmented care and loss to follow-up. For patients with ongoing medical, developmental, or psychosocial complexity, unaddressed social needs may further compound these transition-related risks. In this context, structured documentation of social needs may help identify patients who would benefit from additional transition planning, care coordination, or referral to community-based resources. Furthermore, structured documentation may support continuity of care by ensuring that important information about unmet social needs and existing supports is preserved across care settings during the transition from pediatric to adult care.

### Racial and ethnic differences in Z-code documentation

We observed differences in Z-code documentation by race and ethnicity, with non-White and Hispanic patients demonstrating higher rates of documented Z-codes. These findings may reflect a higher underlying burden of health-related social needs among structurally marginalized populations, consistent with prior literature demonstrating racial, ethnic, and language-based disparities in caregiver-reported unmet social needs [44]. At the same time, differences in documentation may also be influenced by provider-level factors, including greater awareness of social risk in certain populations or implicit biases that affect which patients are more likely to have social needs formally documented. Although we could not distinguish between true differences in social need prevalence and differences in documentation practices, these findings highlight the need for future work using more granular demographic data to better understand potential disparities in both identification and documentation of social needs.

### Are Z-codes the right strategy for documenting social needs?

Our findings raise important questions about the role of ICD-10 Z-codes as a strategy for documenting health-related social needs in pediatric care. Z-codes offer a standardized, system-level mechanism for capturing social risk within electronic health records, enabling aggregation across populations and comparison across clinical settings. However, their nonbillable nature, limited provider awareness, and inconsistent use substantially constrain their effectiveness as a standalone tool.

Importantly, Z-codes are not designed to function as screening instruments. Validated social needs screening tools embedded within clinical workflows are better suited to identifying unmet needs at the point of care, while narrative clinical documentation allows clinicians to capture contextual nuance. In contrast, structured documentation mechanisms such as Z-codes serve a complementary role by enabling health systems to monitor social risk at the population level, allocate resources, and evaluate interventions over time. From this perspective, Z-codes are useful for system-level planning, but may be inadequate without reliable screening processes upstream.

Our findings suggest that clinical environments more likely to engage in routine screening, such as SBHC and pediatric primary care settings, caring for young adults also demonstrate higher rates of structured social needs documentation. Therefore, efforts to improve Z-code utilization might focus less on improving documentation rates alone (for example through targeted provider training) and more on integrating validated standardized screening tools such as the WE CARE instrument [13, 14] into the electronic health record workflow.

Ultimately, while ICD-10 Z-codes are an imperfect tool, they remain a valuable component of a broader, multi-layered strategy for addressing pediatric social needs. When integrated with standardized screening, interdisciplinary care models, and community partnerships, structured documentation can support equitable population health management and inform policy-level decision-making.

### Limitations

This study has several limitations. As a single–health-system analysis, findings may not be generalizable to pediatric populations in other geographic regions or healthcare settings. Furthermore, the study period includes data through the first year of the COVID-19 pandemic in 2020, during which outpatient care delivery and documentation priorities shifted substantially. These disruptions may have influenced both the identification and recording of social needs, limiting applicability to post-pandemic care settings. Additionally, a small number of observations fell outside the expected age range for school-based health centers, likely reflecting real-world scheduling or registration patterns within the electronic health record. These observations were retained, but subgroup findings for these age ranges should be interpreted cautiously.

Sociodemographic variables were constrained by the structure of available electronic health record data. Race, ethnicity, and sex were captured using categories available at the time of data collection and analysis and may not fully reflect patient identity or current federal reporting recommendations, potentially obscuring differences in documentation across demographic subgroups. Furthermore, health plan information and full diagnostic code data were not available, precluding analysis of how these factors may additionally impact Z-code documentation. Finally, we did not adjust for multiple comparisons, which may increase the risk of type I error among subgroup analyses.

### Future directions

Future research should examine Z-code documentation practices across multiple centers using both quantitative and qualitative methods to understand ways to improve utilization. Further work is also needed to evaluate how structured social needs documentation, including but not limited to ICD-10 Z-codes, can be integrated into electronic health records in ways that support accurate identification, longitudinal tracking, and intervention for pediatric patients with unmet social needs.

In addition, the elevated rates of Z-code documentation observed among young adults receiving pediatric care warrant further investigation. Longitudinal studies examining social needs documentation during the transition from pediatric to adult care may help determine whether structured documentation supports care continuity by ensuring that important information about social needs and existing supports is carried across care settings and used to guide transition planning.

Finally, future studies should examine whether improved documentation of pediatric social needs is associated with increased referrals to community-based resources, improved care coordination, and downstream health outcomes, thereby clarifying the role of structured documentation in advancing equitable pediatric care.

## Conclusion

This study adds to the growing body of literature on social needs documentation while providing a unique pediatric perspective. Our results suggest that social needs documentation in the pediatric population remains low overall and is likely unrepresentative of true population needs. While ICD-10 Z-codes offer a standardized mechanism for system-level monitoring, their low uptake underscores the need for further study of how pediatric social needs are identified, documented, and operationalized within clinical workflows. Improving the identification and documentation of social needs in childhood represents a critical opportunity to support early intervention, promote health equity, and inform population-level strategies aimed at improving long-term health outcomes.

## Supplementary Information

Below is the link to the electronic supplementary material.

Supplementary Material 1

## Data Availability

The datasets used and/or analyzed during the current study are available from the corresponding author on reasonable request.

## Abbreviations

HRSN: Health related social needs
ICD: 10–International Classification of Diseases, 10th Revision
SES: Socioeconomic status
