# Supplementary material for: Analyzing pediatric department documentation of patient social needs with ICD-10 Z-codes
Source: BMC Health Serv Res. 2026 Jun 3;26:1087. doi: 10.1186/s12913-026-14758-x (PMC13459765; doi:10.1186/s12913-026-14758-x)
Supplement: Supplementary file 1 [file 12913_2026_14758_MOESM1_ESM.docx]

**SUPPLEMENTAL MATERIAL**

**Supplemental Content 1: Description of ICD-10 Social Needs Related Z-codes**

| Code | Description |
| --- | --- |
| **Z55** | **Problems related to education and literacy** |
| Z55.0 | Illiteracy and low-level literacy |
| Z55.1 | Schooling unavailable and unattainable |
| Z55.2 | Failed school examinations |
| Z55.3 | Underachievement in school |
| Z55.4 | Educational maladjustment and discord with teachers and classmates |
| Z55.5 | Less than a high school diploma |
| Z55.8 | Other problems related to education and literacy |
| Z55.9 | Problems related to education and literacy, unspecified |
|  |  |
| **Z56** | **Problems related to employment and unemployment** |
| Z56.0 | Unemployment, unspecified |
| Z56.1 | Change of job |
| Z56.2 | Threat of job loss |
| Z56.3 | Stressful work schedule |
| Z56.4 | Discord with boss and workmates |
| Z56.5 | Uncongenial work environment |
| Z56.6 | Other physical and mental strain related to work |
| Z56.8 | Other problems related to employment |
| Z56.81 | Sexual harassment on the job |
| Z56.82 | Military deployment status |
| Z56.89 | Other problems related to employment |
| Z56.9 | Unspecified problems related to employment |
|  |  |
| **Z57-Z58** | **Occupational exposure and problems related to physical environment** |
|  |  |
| **Z59** | **Problems related to housing and economic circumstances** |
| Z59.0 | Homelessness |
| Z59.1 | Inadequate housing |
| Z59.2 | Discord with neighbors, lodgers and landlord |
| Z59.3 | Problems related to living in residential institution |
| Z59.8 | Other problems related to housing and economic circumstances |
| Z59.9 | Problem related to housing and economic circumstances, unspecified |
|  |  |
| **Z59.4** | **Lack of adequate food** |
|  |  |
| **Z60** | **Problems related to social environment** |
| Z60.0 | Problems of adjustment to life-cycle transitions |
| Z60.2 | Problems related to living alone |
| Z60.3 | Acculturation difficulty |
| Z60.4 | Social exclusion and rejection |
| Z60.8 | Other problems related to social environment |
| Z60.9 | Problem related to social environment, unspecified |
|  |  |
| **Z62-Z63** | **Problems related to upbringing and primary support group** |
| Z62.1 | Parental overprotection |
| Z62.21 | Child in welfare custody |
| Z62.810 | Personal history of physical and sexual abuse in childhood |
| Z62.812 | Personal history of neglect in childhood |
| Z62.819 | Personal history of unspecified abuse in childhood |
| Z62.820 | Parent-biological child conflict |
| Z62.821 | Parent-adopted child conflict |
| Z62.822 | Parent-foster child conflict |
| Z62.890 | Parent‒child estrangement NEC |
| Z62.898 | Other specified problems related to upbringing |
| Z62.9 | Problem related to upbringing, unspecified |
| Z63.0 | Problems in relationship with spouse or partner |
| Z63.32 | Other absence of family member |
| Z63.4 | Disappearance and death of family member |
| Z63.5 | Disruption of family by separation and divorce |
| Z63.6 | Dependent relative needing care at home |
| Z63.72 | Alcoholism and drug addiction in family |
| Z63.79 | Other stressful life events affecting family and household |
| Z63.8 | Other specified problems related to primary support group |
| Z63.9 | Problem related to primary support group, unspecified |
|  |  |
| **Z64-Z65** | **Problems related to certain psychosocial circumstances** |
| Z64.0 | Problems related to unwanted pregnancy |
| Z64.1 | Problems related to multiparity |
| Z64.4 | Discord with counselors |
| Z65.1 | Imprisonment and other incarceration |
| Z65.3 | Problems related to other legal circumstances |
| Z65.4 | Victim of crime and terrorism |
| Z65.5 | Exposure to disaster, war and other hostilities |
| Z65.8 | Other specified problems related to psychosocial circumstances |
| Z65.9 | Problem related to unspecified psychosocial circumstances |
|  |  |
| **Z74.8 & Z91.89** | **Problems related to transportation** |
| Z74.8 | Assistance needed with transportation |
| Z91.89 | Lack of access to transportation |
